# Case report: Fatal ischemic stroke induced by unruptured traumatic intracranial vertebral artery dissection

**DOI:** 10.3389/fneur.2023.1202698

**Published:** 2023-09-12

**Authors:** Shuheng Wen, Kana Unuma, Motoki Inaji, Yohsuke Makino, Shutaro Nagano, Kazuki Harada, Nobutaka Arai, Koichi Uemura

**Affiliations:** ^1^Department of Forensic Medicine, Graduate School of Medical and Dental Sciences, Tokyo Medical and Dental University (TMDU), Tokyo, Japan; ^2^Department of Neurosurgery, Tokyo Medical and Dental University (TMDU), Tokyo, Japan; ^3^Department of Forensic Medicine, Graduate School of Medicine, The University of Tokyo, Tokyo, Japan; ^4^Department of Forensic Medicine, National Defense Medical College, Saitama, Japan

**Keywords:** intracranial vertebral artery dissection, ischemic stroke, traumatic intracranial artery dissection, forensic autopsy, shower embolism

## Abstract

Intracranial vertebral artery dissection (IVAD) is rare and potentially fatal due to the risk of secondary subarachnoid hemorrhage once ruptured. Unruptured traumatic IVAD is even rarer and can result in ischemic stroke, yet mostly benign when timely diagnosed. Herein, we present an uncommon case of a patient who underwent a fatal ischemic stroke induced by unruptured traumatic IVAD. The patient was symptomatic soon after being physically assaulted but left untreated until acute deterioration for multiple brain infarctions occurred, secondary to IVAD-induced cerebellar stroke. Fifteen days later, he died, regardless of an urgently performed thrombectomy. Multiple serial histologic examinations revealed an unruptured dissection of the intracranial vertebral artery with a slit-like tear of the intimal and medial layers, considered to be the culprit lesion. The 15-day prolonged onset of stroke was rare in traumatic IVADs. Furthermore, the slit-like tear of the intimal layer in our case may support the initial intimal laceration hypothesis for VAD pathogenesis. Since limited pathohistological information is available regarding ischemic IVAD, we believe this rare case will be beneficial in understanding the pathophysiology of ischemic IVAD.

## 1. Introduction

Intracranial vertebral artery dissection (IVAD) is a rare condition that occurs when blood enters the medial layer following a spontaneous or traumatic disruption of the intimal layer (1, 2). Risk factors and predisposing conditions of IVAD include hypertension, cervicocerebral trauma, connective tissue disease, and congenital vascular malformations (3–6). The nondominant intracranial vertebral artery and the portion around the posterior inferior cerebellar artery (PICA) orifice are more frequently involved in IVAD (7). IVAD can result in ischemic stroke or subarachnoid hemorrhage (8, 9). Compared to the outcome of the hemorrhagic type, ischemic IVAD is considered to have a favorable outcome with a low risk of subsequent hemorrhage (1, 10, 11); however, it is an important cause of cerebellar and brainstem infarctions, especially in young adults (1, 12, 13). A timely and accurate diagnosis is essential in this situation because the mass effect of the infarcted cerebellum, which is located in the tightly constrained posterior fossa, can lead to devastating complications including brainstem compression and obstructive hydrocephalus (3, 13).

Though aspects like epidemiology; pathophysiology; clinical, radiological, as well as pathohistological characteristics; and management of subarachnoid hemorrhage induced by ruptured IVAD has been extensively described and studied in literature (3, 10, 14), limited information is available about ischemic strokes related to unruptured IVAD subsequent to a traumatic assault, partially owing to its extremely low incidence (7). Herein, we present a rare case of a man in his forties who developed symptomatic traumatic IVAD and secondary cerebellar stroke after being physically assaulted. The patient acutely deteriorated 15 days after the event and eventually died of multiple secondary brain infarctions. In this report, the death mechanism of this rare case was discussed. We believe it is the first case report showing traumatic unruptured IVAD-induced fatal ischemic stroke with detailed autopsy, radiological, and histological findings, which should be beneficial in understanding the pathophysiology of vertebral artery dissection.

## 2. Case description

A man in his forties was assaulted by multiple perpetrators who kicked and hit all over his body, mainly the head and neck area. Although the patient complained of dizziness and pain at the back of his head to the police and emergency crews, he refused hospital admission and left the crime scene on his own. In the following 4 days, the man visited a clinic for pain on the medial side of his left knee but received no medication. According to his friend, who met him 9 days after the assault, the man limped and complained of dizziness at that time.

**Figure 1:**
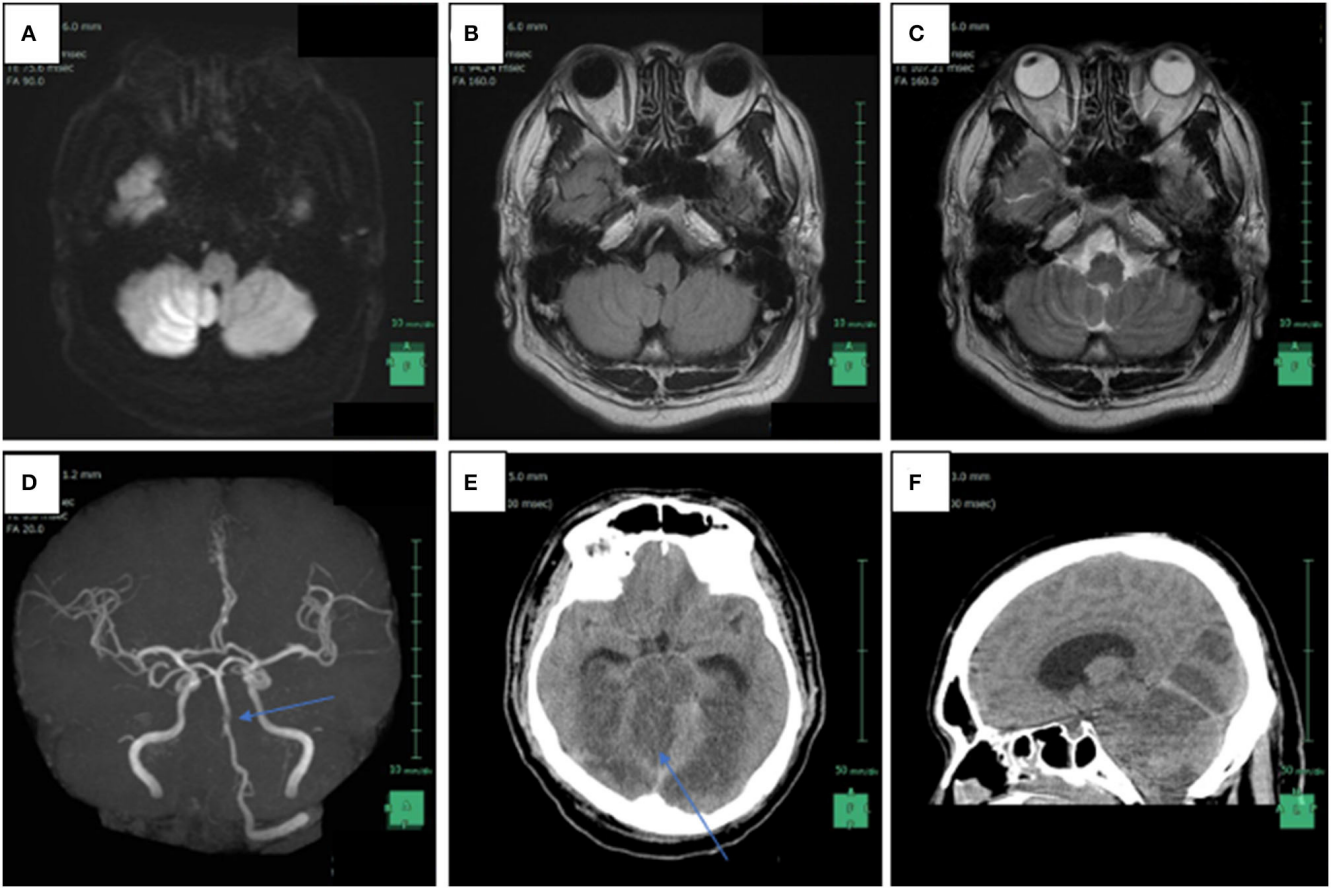
Radiological findings of patient 15 days after the violent assault. **(A)** Axial head magnetic resonance imaging (MRI) in diffusion weighted imaging showed increased signal intensity in the right intracranial vertebral artery (IVA) and a high intensity area in the bottom of the right cerebellar hemisphere, which is compatible with the posterior inferior artery (PICA) territory; **(B)** FLAIR weighted imaging also revealed increased signal intensity in the right IVA; **(C)** T2-weighted axial head MRI image reveals the absence of flow void in the right IVA; **(D)** non-contrast enhanced magnetic resonance angiography shows disappearance of signals from the right IVA and a part of the basilar artery (arrow); **(E)** axial head computed tomography scanned 1 day after the MRI reveals low-density areas in the brainstem, the bilateral occipital lobes, and the right cerebellum, as well as brain swelling; **(F)** sagittal head computed tomography reveals tonsillar herniation and supratentorial ventricular enlargement.

On the 15th day after the assault, the man called an ambulance as he was experiencing vertigo, nausea, and pain at the back of his head. Although he was unable to walk independently, he was conscious at the time of his visit to the hospital. However, 2 h later, while he was undergoing a magnetic resonance imaging, his status suddenly deteriorated, and he showed decerebrate posturing and loss of consciousness. Radiographic findings suggested right intracranial vertebral artery (IVA) occlusion, multiple brain infarctions including brainstem, bilateral occipital lobes, and the right cerebellum, as well as brain swelling and tonsillar herniation (Figure 1). Despite of an instantly conducted percutaneous thrombectomy, the patient failed to retrieve his brainstem function and was confirmed dead 9 days after being admitted. Given that the IVA lesion may result from the premorbid violent assault the patient experienced, the neurosurgeon contacted the police. To determine the death mechanism and the causal relationship between the premorbid trauma and the death, a judicial autopsy was conducted 37 h after his death entrusted by the police.

The patient had a history of gonorrhea, attention deficit hyperactivity disorder, hypertension, diabetes mellitus, hyperuricemia, and hyperlipidemia. He was prescribed with aripiprazole, bisoprolol fumarate, eszopiclone, and methylphenidate.

## 3. Autopsy and laboratory findings

### 3.1. Autopsy findings

**Figure 2:**
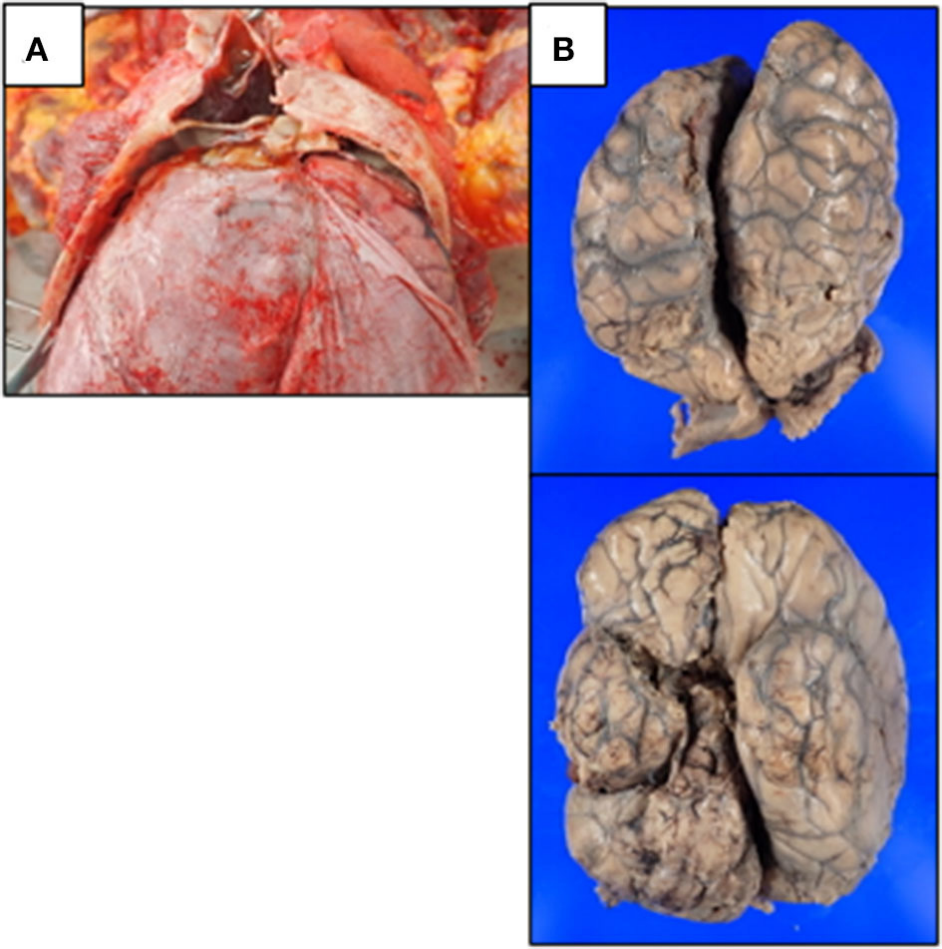
Macroscopic findings during autopsy. **(A)** Partially healed fractures of the frontal bone and an old epidural hematoma were observed. **(B)** The brain of the deceased man was swollen and soft with flattened convolutions; No apparent hemorrhage was found in the subarachnoid space.

The deceased man was 171 cm tall and weighed 93.1 kg (body mass index: 31.84 kg/m^2^). He had facial trauma from the injury, including a black eye, but the facial injuries had healed by the time of autopsy. Internal examination revealed over 10 spotted subcutaneous and subgaleal hemorrhages on the posterior and right parts of the head. The left and right bulbar conjunctivae were pale reddish and edematous with extensive subconjunctival hemorrhage. Partially healed fractures of the frontal bone and an epidural hematoma were visible (Figure 2A). There was no hematoma in the subdural space, nor any fractures in the skull base or cervical spine. The brain, weighted 1,450 g, was swollen and soft with flattened convolutions. No apparent hemorrhage was found in the subarachnoid or subpial space (Figure 2B). There was also no evident atherosclerosis in the arteries of the base of the brain. No apparent injury to the cervical region was observed. Internal examination revealed hemorrhage in the connective tissue between the foramen magnum and the posterior of the upper cervical vertebra. Considering the clinical diagnosis of intracranial vertebral artery occlusion and multiple brain infarctions, the circle of Willis from the V2 segments of the bilateral vertebral arteries to the bilateral anterior cerebral arteries was carefully removed for histological examination (Supplementary Figure 1).

The heart weighed 570 g. The ventricular wall was thick, and hypertensive cardiac hypertrophy was observed. No thrombus or apparent narrowing was observed among the coronary arteries. No significant pathological changes were observed in any other organ. Scattered purple-reddish skin discolorations and subcutaneous hemorrhages were found in the limbs with the largest one sized 9.5 × 0.3 cm and 2.3 × 0.6 cm, respectively. However, there was no pathological change observed in the left knee.

### 3.2. Histological findings

**Figure 3:**
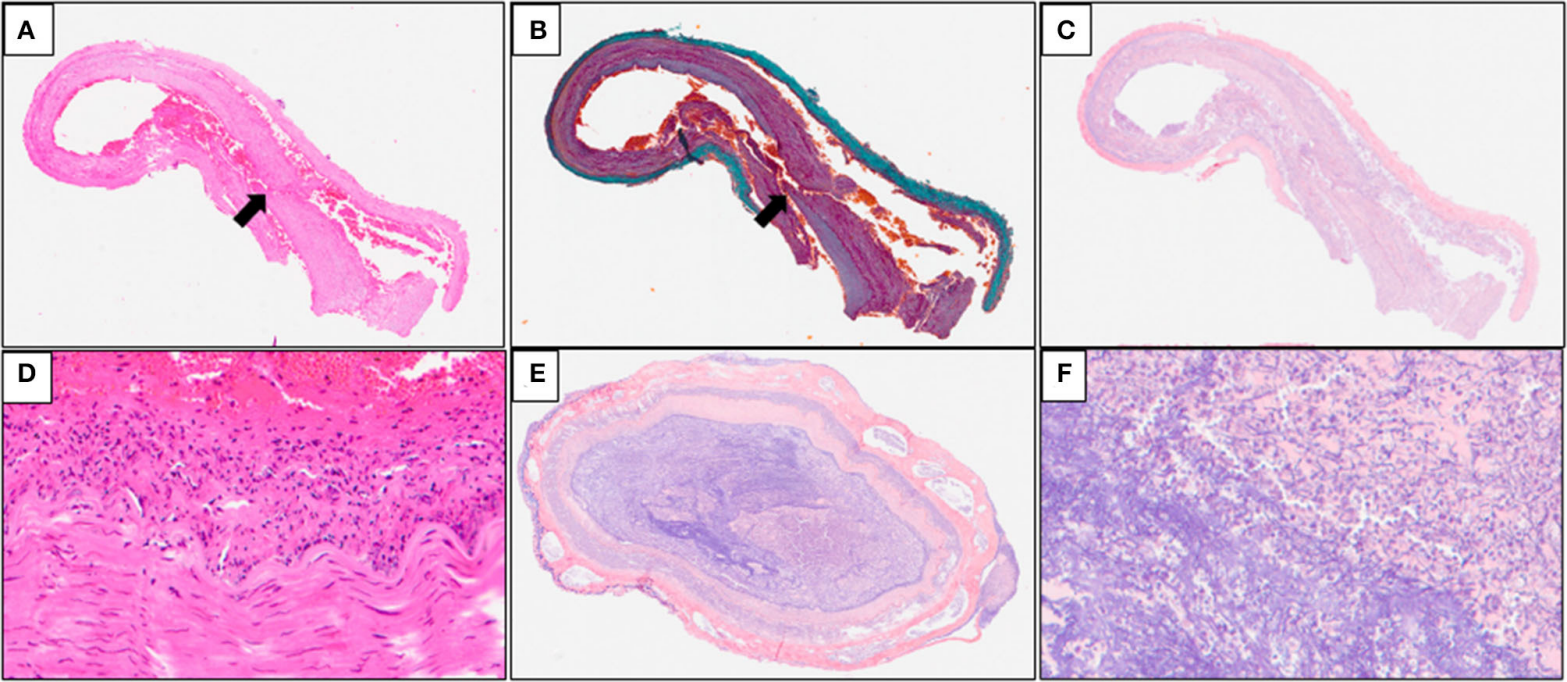
Microscopic findings of the arteries of the brain base. **(A, B)** Hematoxylin and eosin (H&E) and Elastica Masson-Goldner (EMG) stainings of the brain base artery reveals a slit-like tear in the intimal and medial layers of the right IVA V4 (arrows) segment with blood infiltration between the medial and adventitial layers; **(C)** Phosphotungstic acid hematoxylin (PTAH) staining of the samples does not reveal any apparent disruption of the medial layer and fibrin thrombus at the site of the dissection; **(D)** inflammatory cell infiltrates are seen on the intimal side; **(E, F)** PTAH staining of the adjacent right IVA samples revealed complete occlusion caused by fibrin thrombi, mild inflammatory infiltrates in the intimal layer, and expansion of the vasa vasorum.

**Figure 4:**
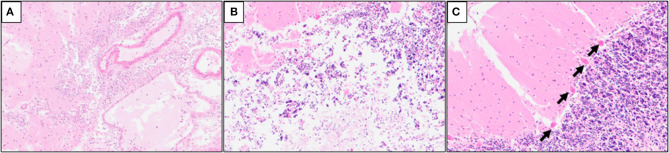
Hematoxylin and eosin (H&E) staining of the cerebellum samples. **(A)** Mild inflammatory cell infiltrates in the subarachnoid space of the right occipital lobe; **(B)** mild macrophage infiltration in the infarct lesions of the right cerebellum; **(C)** ischemic changes of the Purkinje cells (arrows) in the right cerebellum.

Multiple serial histologic sections of collected brain base artery samples were examined microscopically following hematoxylin and eosin (H&E), Elastica Masson-Goldner (EMG), and phosphotungstic acid Hematoxylin (PTAH) stainings. A slit-like tear of the intimal and medial layers of the right IVA V4 segment with blood infiltration between the medial and adventitial layers, was revealed by H&E and EMG stainings, indicating an unruptured right IVAD (Figures 3A, B). No apparent fibrin thrombus at the site of dissection (Figure 3C) was observed with PTAH staining; however, inflammatory cell infiltrates were observed on the intimal side (Figure 3D). Furthermore, samples of the adjacent right IVA showed complete occlusion because of fibrin thrombi, mild infiltration of inflammatory cells into the intimal layer, and expansion of the vasa vasorum (Figures 3E, F). Fibrin thrombi were also found in the bilateral anterior cerebral arteries, the bilateral middle cerebral arteries, the bilateral posterior cerebral arteries, and the basilar artery. Moreover, mild infiltration of inflammatory cells was observed in the subarachnoid space of the right occipital lobe (Figure 4A). The cerebellum showed extensive infarct lesions and mild inflammatory infiltration (Figures 4B, C). No other ruptures or lesions were observed in the cerebral arteries.

### 3.3. Laboratory findings

Liquid chromatography-mass spectrometry revealed that relatively low concentrations of acetaminophen, aripiprazole, and nicotine metabolites were present in the patient's blood sample. Ethanol was not detected in the blood samples.

## 4. Discussion

Since the autopsy and radiographic findings indicated right stenotic IVAD in the V4 segment and multiple brain infarctions affecting the cerebellum, brainstem, and cerebrum, we surmised that the fibrin thrombus formed after dissection at the site of lesion was carried away by the blood current and redistributed into the downstream brain arteries, causing secondary brain infarctions in related territories. The brainstem compression could have been a result of either the mass effect that occurred following the right cerebellar infarction, which developed owing to occlusion of the PICA, or a direct brainstem infarction induced by the drifted fibrin thrombus, or a combination of these two circumstances that led to acute deterioration of brain death in the deceased man. Although intracranial artery dissection can be spontaneously caused by atherosclerosis, vasculitis, or connective tissue disorders (3), the isolated artery dissection and the absence of vascular abnormality as well as systematic inflammatory disorder findings in this case disfavored the explanation of spontaneous artery dissection. Moreover, the slit-like tear of the intimal and medial layers of the involved vertebral artery was consistent with the findings of traumatic rupture of the intracranial vertebral artery. It has been indicated that the intimal and medial tear appears to be oblique in traumatic rupture of the intracranial vertebral artery, while the intimal tear occurs at the recessed vasculature with massive defect of medial structure in non-traumatic IVAD (15). Further considering the patient's complaints and his permanent symptoms after the assault, we believe that the IVAD had already developed at the scene and that the exact onset of IVAD-induced cerebellar stroke was earlier than the acute deterioration. Consequently, the cause of death was determined to be multiple brain infarctions and hypoxic-ischemic encephalopathy secondary to a stenotic IVAD due to an external cause. Management of ischemic IVAD involves conservative treatments like anticoagulants and follow-up stroke prevention, yet interventional treatments like stent-assisted coiling or vessel sacrifice can be optional since the optimum management strategy remains debatable (3, 16). Although the prevention of multiple cerebral infarctions following ischemic IVAD can be expected with timely medical treatment, in this case, it is difficult to evaluate the risk of the development of secondary subarachnoid hemorrhage. In any case, a timely diagnosis can be challenging in unruptured IVADs because of the vague onset of signs and symptoms, similar to our case (17). With the benefit of hindsight and the absence of positive findings in the knee, one can probably realize that the initial presentation of contralateral knee pain and gait disruption of the patient following the assault, was likely attributed to the involvement of PICA.

It is worth noticing that the 15-day prolonged onset of stroke was not commonly seen in traumatic IVADs. We speculated that two factors were implicated in the delayed onset in our case. It is believed that the lack of medial and external elastic fibers in the intradural portion and weaker supporting tissues make the V4 segment of the intracranial vertebral artery more susceptible to subadventitial dissection (7, 18, 19). On one hand, the uncommon presence of internal and external elastic lamina in the V4 segment of this patient may have provided a stronger structure and rendered the vessel more resistant to complete rupture than normal (Figure 2B). On the other hand, certain drug prescribed to the patient has an unexpected antithrombotic effect which may protract the stroke onset. Bisoprolol, besides its antihypertensive property, has been reported to increase permeability of plasma fibrin clots and to reduce clot lysis time (20). Jones et al. reported a similar delayed onset case where a woman in her thirties developed IVAD-induced cerebellar stroke and hydrocephalus 2–3 weeks after neck manipulation. With only unilateral intracranial vertebral artery involved, this patient was uneventfully discharged after receiving mannitol treatment and ventriculostomy for approximately 9 days (21). On the contrary, in this case, fibrin thrombi were found in multiple main cerebral arteries including the right intracranial vertebral artery, the bilateral anterior cerebral arteries, the bilateral middle cerebral arteries, the bilateral posterior cerebral arteries, and the basilar artery. The involvement of the basilar artery and multiple inclusive infarct lesions are independent unfavorable outcome predictors for intracranial artery dissections, which is in accordance with the tragic outcome in our case (3, 22).

The pathophysiology of vertebral artery dissection is still unknown, especially for the ischemic type (23), not only because of the extremely low incidence of IVAD, but also because co-ordination of neurologists, neurosurgeons, radiologists, and forensic pathologists is required for insights into the pathophysiology (3). Multiple mechanisms of VAD onset have been proposed, including initial intimal laceration and rupture of the vasa vasorum (24). The slit-like tear of the intimal layer in our rare case seems to suit the initial intimal laceration mechanism.

In summary, we present a rare case of fatal ischemic stroke induced by unruptured traumatic IVAD with detailed autopsy, histopathological, and radiological findings. Since limited pathohistological information is available regarding ischemic IVAD, we hope this case will be beneficial in understanding the pathophysiology of ischemic IVAD.

## Data availability statement

The datasets presented in this study can be found in online repositories. The names of the repository/repositories and accession number(s) can be found in the article/Supplementary material.

## Ethics statement

Ethical review and approval was not required for the study on human participants in accordance with the local legislation and institutional requirements. Written informed consent from the patients/ participants or patients/participants' legal guardian/next of kin was not required to participate in this study in accordance with the national legislation and the institutional requirements. Written informed consent was obtained from the individual(s) and/or minor(s)' legal guardian/next of kin for the publication of any potentially identifiable images or data included in this article.

## Author contributions

Conception and diagnosis of the case: KaU and NA. Performed the autopsy: KaU. Analysis of the data: SW, KaU, MI, YM, KH, and NA. Writing of the manuscript: SW, KaU, and MI. Critical revision of the manuscript: SN, KH, and KoU. All authors contributed to the article and approved the submitted version.
