# Supplementary material for: Case report: Fatal ischemic stroke induced by unruptured traumatic intracranial vertebral artery dissection
Source: Front Neurol. 2023 Sep 12;14:1202698. doi: 10.3389/fneur.2023.1202698 (PMC10536137; doi:10.3389/fneur.2023.1202698)
Supplement: Supplementary file 1 [file Data_Sheet_1.docx]

Supplementary Material

Case Report: Fatal Ischemic Stroke Induced by Unruptured Traumatic Intracranial Vertebral Artery Dissection

**Shuheng Wen, Kana Unuma*, Motoki Inaji, Yohsuke Makino, Shutaro Nagano, Kazuki Harada, Nobutaka Arai, Koichi Uemura**

**Correspondence:** Corresponding Author: Kana Unuma, unumlegm@tmd.ac.jp

# Supplementary Figure

##

**Supplementary Figure 1.** Multiple serial histologic examinations of the circle of Willis from the V2 segment of the bilateral vertebral arteries to the bilateral anterior cerebral arteries. The prepared blocks are also shown.
